# Clinicopathological features, treatment patterns, and prognosis of squamous cell carcinoma of the breast: an NCDB analysis

**DOI:** 10.1186/s12885-018-5212-x

**Published:** 2019-01-08

**Authors:** Liling Zhu, Kai Chen

**Affiliations:** 1 Guangdong Provincial Key Laboratory of Malignant Tumor Epigenetics and Gene Regulation, Sun Yat-Sen Memorial Hospital, Sun Yat-Sen University, Guangzhou, Guangdong China; 2 Department of Breast Surgery, Breast Tumor Center, Sun Yat-sen Memorial Hospital, Sun Yat-sen University, 107 Yanjiang West. Road, Guangzhou, 510120 People’s Republic of China; 3 Department of Biostatistics, School of Public Health, Yale University, 300 George Suit 503, New Haven, CT 06511 USA

**Keywords:** Breast cancer, Squamous cell carcinoma, Infiltrating ductal carcinoma, Survival, Endocrine therapy, Chemotherapy

## Abstract

**Background:**

Squamous cell carcinoma (SCC) of the breast is a rare malignancy. The clinicopathological features, treatment patterns and prognosis of SCC of the breast is still unclear.

**Methods:**

In this study, we performed a 1:4 SCC-IDC (infiltrating ductal carcinoma) matching analysis of patients diagnosed between 2004 and 2014, using the data from the national cancer database. We used Chi-square test to compare the clinicopathological features and treatment patterns between SCC (*n* = 686) and IDC (*n* = 2744) patients. We used Kaplan-Meier analysis and Cox-regression to estimate the survival of SCC and IDC patients.

**Results:**

We observed that SCC patients are more likely to have T3–4, grade III, and ER negative diseases, when compared to IDC patients. Breast conserving surgery (BCS) (58.3% vs 65.4%, *p* = 0.048), as well as radiotherapy after BCS (65.3% vs. 83.0%, *p* < 0.001), was less performed in SCC patients. Among low-risk patients, chemotherapy was used more often for SCC patients (42.9%) than for IDC (18.7%) patients (*p* = 0.002). In HR-positive patients, endocrine therapy was used less often for SCC patients (51.6%) than for IDC patients (70.5%) (*p* < 0.001). SCC (vs. IDC) was associated with no responses to neoadjuvant chemotherapy (20% vs. 5.05%, *p* = 0.019). Adjusted analysis confirmed that SCC (vs. IDC) was associated with worse OS (HR = 1.40, 95%CI 1.17–1.67, *P* < 0.01), after a median follow-up of 58.3 months. In SCC patients, HR status is not prognostic of OS, but endocrine therapy was significantly associated with improved OS in HR-positive SCC patients.

**Conclusions:**

We conclude that SCC is associated with poorer clinicopathological features, no responses to neoadjuvant chemotherapy and worse clinical outcomes than IDC. The treatment patterns for SCC and IDC are different. Endocrine therapy is necessary for HR-positive SCC patients.

**Electronic supplementary material:**

The online version of this article (10.1186/s12885-018-5212-x) contains supplementary material, which is available to authorized users.

## Background

Squamous cell carcinoma (SCC) of the breast is a rare malignancy that accounts for < 0.2% of all breast cancers [1]. Diagnosis of SCC can be made when a predominance (> 90%) of areas with squamous cells is noticed at histology examinations [2]. The origin of the SCC component is still an unanswered question. A recent study [3] showed that SCC and its adjacent infiltrating ductal carcinoma (IDC) component shared the same origin, but their transcription landscape [4] and driven-pathways [5] are different. Thus, whether the differing histology of SCC may result in different biological behavior, different treatment patterns and prognosis is not clear. Most of the studies [1, 6] are limited significantly by their small sample size, due to its rarity. Therefore, a national cancer database remains as the only choice to provide adequate sample size to investigate SCC of the breast.

The national cancer database (NCDB) is a hospital-based database that covers approximately 70% of cancer patients in the United States [7]. The participating centers are required to submit data to the database. In this study, we used the NCDB to compare the clinicopathological features, treatment patterns and prognoses of IDC and SCC patients. We hypothesized that SCC (vs. IDC) was associated with poor clinicopathological characteristics, different treatment patterns, and worse survival. We also performed an exploratory analysis of the benefits of systemic therapies for SCC patients.

## Methods

We searched the NCDB for eligible patients using the inclusion and exclusion criteria below:

### Inclusion

1. Female patients with pathologically confirmed breast cancer,
2. Patients who were diagnosed between 2004 and 2014, and
3. Diagnoses of SCC of the breast (code 8070–8078), and IDC (code 8500).

### Exclusion criteria

1. Patients with prior diagnoses of malignant tumors and
2. A number of follow-up months equal to 0.

A total of 686 SCC patients and 1,211,403 IDC patients were identified from the database. Given the huge discrepancy of the amount of the SCC and IDC patients, we performed a 1:4 SCC-IDC matching on the following factors: Year of diagnosis (2004–2014), Facility type (Community Cancer Program, Comprehensive Community Cancer Program, Academic/Research Program, Integrated Network Cancer Program, Unknown), Facility location (New England, Middle Atlantic, South Atlantic, East North Central, East South Central, West North Central, West South Central, Mountain, Pacific), city type (Metropolitan, Non-metropolitan/Unknown), type of insurance (Not insured, Private insurance, Medicaid, Medicare, other Government, Unknown.). There were 686 SCC and 2744 IDC patients being selected as the final cohort for analysis. This study was an epidemiological study using de-identified data from the NCDB database. Therefore, consent for patient participation and study publication was not required. The study approval was waived by the ethical committee of Yale University and Sun Yat-sen Memorial Hospital.

The following data were collected for each patient: the year of diagnosis, age, race, Charlson-Deyo score, tumor grade, lymphovascular invasion, T-stage, N-stage, histology, estrogen receptor (ER) status, progesterone receptor (PR) status, HER2 status, primary surgery categorization, radiation therapy (RT), chemotherapy, neoadjuvant chemotherapy, response to chemotherapy, endocrine therapy, survival month and OS status. Patients were categorized into three age groups based on their ages at diagnosis (≤50 yrs., 50–60 yrs., > 60 yrs). We used this cut-off because the median age was close to 60 yrs. in our study population and because 50 yrs. is the usual cut-off age for premenopausal and post-menopausal women. Histology was divided into two categories, namely, IDC and SCC.

### Statistical analysis

We conducted a descriptive analysis of the baseline clinicopathological features of the included patients and used the Chi-square test to compare the characteristics of the patients with different histologies. The median follow-up time was calculated as the median observed survival time of the entire population. OS was measured as the time from diagnosis to death due to any cause. The cumulative OS rates were estimated using Kaplan-Meier analysis. We used a Cox regression model to screen for prognostic factors of OS. We tested the proportional hazards assumption by plotting the scaled Schoenfeld residuals of all coefficients over time and found no violations. All *P-*values were two-sided. *P-*values less than 0.05 were considered statistically significant. Statistical analysis was performed using Stata/MP, version 13.0 (StataCorp LP, College Station, TX, USA).

## Results

A total of 3430 patients (IDC: 2744; SCC: 686), with a median age of 61 years, were included in this study. The clinicopathological features are listed in Table 1. In general, SCC is associated with poorer clinicopathological features. A total of 23.1% of the SCC patients had T3–4 disease, whereas only 4% of the IDC patients had T3–4 disease. The proportions of grade III disease were 61.06 and 39.10% for the SCC and IDC patients, respectively. Additionally, the proportions of ER-negative tumors were 74.91 and 21.95% for the SCC and IDC patients, respectively. The distribution of N-stage was similar between SCC and IDC patients.

**Table 1:** Clinicopathological features of study population

	Histology				*P*
	Infiltrating Ductal Carcinoma		Squamous Cell Carcinoma		
	N	%^a^	N	%^a^	
Race					
White	2259	83.33	540	79.65	< 0.01
African American	343	12.65	119	17.55	
Others	109	4.02	19	2.80	
Unknown	33		8		
Charlson-Deyo Score					
0	2346	85.50	555	80.90	0.02
1	328	11.95	104	15.16	
2	54	1.97	20	2.92	
3	16	0.58	7	1.02	
Grade					
I	476	18.90	62	11.33	< 0.01
II	1058	42.00	151	27.61	
III	985	39.10	334	61.06	
Unknown/IV	225		139		
Lymphovascular Invasion					
Absence	768	79.67	185	85.65	0.04
Present	196	20.33	31	14.35	
Not Applicable	1780		470		
T-Stage					
T0-T1	1441	52.51	145	21.14	< 0.01
T2	502	18.29	178	25.95	
T3	67	2.44	100	14.58	
T4	45	1.64	58	8.45	
Tx	689	25.11	205	29.88	
N-Stage					
N0	1373	50.04	331	48.25	0.228
N1	381	13.88	85	12.39	
N2	115	4.19	22	3.21	
N3	56	2.04	16	2.33	
Nx	819	29.85	232	33.82	
M-Stage					
M0	2250	82.00	490	71.43	< 0.01
M1	49	1.79	23	3.35	
Mx	445	16.22	173	25.22	
Estrogen Receptor					
Negative	569	21.95	421	74.91	< 0.01
Positive	2023	78.05	141	25.09	
Unknown	152		124		
Progesterone Receptor					
Negative	843	32.69	491	87.84	< 0.01
Positive	1736	67.31	68	12.16	
Unknown	165		127		
HER2^b^					
Negative	829	78.2	207	87.7	< 0.01
Borderline	31	2.9	2	0.9	
Positive	200	18.9	27	11.4	
Unknown	156		68		
Breast Surgery					
No_Surgery	167	6.10	90	13.16	< 0.01
Breast-conserving surgery	1561	56.99	246	35.96	
Mastectomy	1010	36.87	348	50.88	
Surgery (Types_Unknown)	1	0.04	0	0.00	
Unknown	5		2		
Radiation Therapy					
No	1119	41.23	373	54.93	< 0.01
Yes	1595	58.77	306	45.07	
Unknown	30		7		
Chemotherapy					
None	1506	58.28	294	46.08	< 0.01
Single-Agent Chemotherapy	44	1.70	23	3.61	
Multiagent Chemotherapy	1034	40.02	321	50.31	
Unknown	160		48		
Neoadjuvant chemotherapy					
No	1884	89.89	447	83.86	< 0.01
Yes	212	10.11	86	16.14	
Unknown	91		14		
Endocrine Therapy					
No	1110	42.53	567	85.91	< 0.01
Yes	1500	57.47	93	14.09	
Unknown	134		26		

^a^Percentages were calculated based on the available data

^b^Only patients after 2010 were used for analysis of HER2 status

### Different treatment patterns between SCC and IDC patients

In patients with T1–2 stages who did not received neoadjuvant chemotherapy, there were 58.3% (120/206) vs. 65.4% (890/1362) of the SCC and IDC patients received BCS (*P* = 0.048) respectively. Among the patients with BCS (*N* = 1791, 16 patients with unknown RT status were excluded.), 65.3 and 83.0% of the SCC and IDC patients received RT (*P* < 0.01), respectively. The use of RT in node-positive patients with mastectomies, were similar in SCC and IDC patients (42.1% (37/88) vs. 48.4%(166/343), *P* = 0.287).

In patients with favorable prognosis (hormone receptor (HR)-positive, HER2-negative and node-negative), chemotherapy was performed in 42.9% (12/28) and 18.7% (80/427) of the SCC and IDC patients, respectively (*P* = 0.002). In the HR-positive, node-negative patients who were diagnosed before 2010 (when the HER2 status was unknown), there were 51.6% (32/62) and 24.9% (248/997) of the SCC and IDC patients who had received chemotherapy, respectively (*P* < 0.001). Among the patients with HR-positive disease, endocrine therapy was performed in 51.6% (79/153) and 70.5% (1446/2050) of the SCC and IDC patients, respectively (*P* < 0.001).

### Response to neoadjuvant chemotherapy

In this study, there were 298 patients with known history of neoadjuvant chemotherapy, and 129 of them had clear information about treatment responses (CR, PR, CR/PR, No response). SCC (vs. IDC) was significantly associated with no responses to neoadjuvant chemotherapy (20% vs. 5.05%, *P* = 0.019) (Table 2).

**Table 2:** Response to neoadjuvant chemotherapy by histology

Category 1									
Histology	CR		PR, CR/PR		No response		Total		P*
	N	%	N	%	N	%	N	%	
IDC	35	35.35	59	59.6	5	5.05	99	100	0.042
SCC	8	26.67	16	53.33	6	20	30	100	
Category 2									
Histology	Response (CR, PR, CR/PR)				No response		Total		P*
	N		%		N	%	N	%	
IDC	94		94.95		5	5.05	99	100	0.019
SCC	24		80		6	20	30	100	

*CR* Complete Response, *PR* Partial Response, *IDC* Infiltrating Ductal Carcinoma, *SCC* Squamous Cell Carcinoma

*Fisher Exact test

### Survival analysis

With a median follow-up time of 58.3 months, the respective 5-yr and 10-yr OS were 62.1 and 50.6% for the SCC patients, and 83.0 and 69.5% for the IDC patients, respectively (*P* < 0.001). SCC (vs. IDC) was associated with poorer OS in univariate analysis (HR = 2.39, 95%CI 2.06–2.77, *P* < 0.001), and in multivariate (HR = 1.40,95%CI 1.17–1.67, *P* < 0.001) analysis after adjusting for age, race, comorbidity, T-stage, N-stage, M-stage, ER, PR, tumor grade, LVI, surgery, endocrine therapy, chemotherapy and RT (Table 3, & Additional file 1: Figure S1). In patients who were diagnosed after 2010, SCC (vs. IDC) was still associated with poorer OS (HR = 1.57, 95%CI 1.11–2.21, *P* = 0.011), after adjusting for the above variables, as well as LVI and HER2 status.

**Table 3:** Univariate and multivariate analysis of prognostic factors of OS

Variables	Univariate analysis		Multivariate analysis	
	HR(95%CI)	*P*	HR(95%CI)	*P*
Age				
< =50	1		1	
50–60	1.13 (0.89–1.44)	0.301	1.10 (0.86–1.40)	0.445
> 60	2.25 (1.86–2.74)	< 0.001	2.11 (1.72–2.60)	< 0.001
Race				
White	1		1	
African American	1.51 (1.26–1.80)	< 0.001	1.05 (0.87–1.27)	0.576
Others	0.43 (0.24–0.77)	0.004	0.48 (0.27–0.85)	0.012
Unknown	0.53 (0.24–1.19)	0.126	0.49 (0.22–1.10)	0.082
Comorbidity score				
Score 0	1		1	
Score 1	2.17 (1.85–2.54)	< 0.001	1.65 (1.40–1.95)	< 0.001
Grade				
I	1		1	
II	1.20 (0.94–1.54)	0.149	0.98 (0.76–1.26)	0.877
III	2.05 (1.62–2.58)	< 0.001	1.35 (1.05–1.74)	0.02
Unknown/IV	1.86 (1.39–2.49)	< 0.001	1.05 (0.77–1.42)	0.767
T-stage				
T0-T1	1		1	
T2	2.24 (1.86–2.69)	< 0.001	1.65 (1.35–2.03)	< 0.001
T3–4	5.63 (4.60–6.90)	< 0.001	2.75 (2.14–3.55)	< 0.001
Tx	1.25 (1.04–1.51)	0.017	0.62 (0.45–0.85)	0.003
N-stage				
N0	1		1	
N1	1.82 (1.49–2.22)	< 0.001	1.77 (1.43–2.18)	< 0.001
N2–3	3.61 (2.90–4.49)	< 0.001	2.18 (1.69–2.83)	< 0.001
Nx	1.12 (0.94–1.32)	0.197	0.95 (0.70–1.30)	0.764
M-stage				
M0	1		1	
M1	8.47 (6.42–11.17)	< 0.001	4.50 (3.29–6.16)	< 0.001
Mx	2.04 (1.74–2.39)	< 0.001	1.90 (1.53–2.36)	< 0.001
Histology				
Infiltrating Ductal Carcinoma	1		1	
Squamous Cell Carcinoma	2.39 (2.06–2.77)	< 0.001	1.37 (1.15–1.64)	< 0.001
Lymphovascular invasion^a^				
Absence	1		Not included	
Presence	1.91 (1.37–2.67)	< 0.001		
Not applicable/Unknown	2.18 (1.63–2.91)	< 0.001		
Estrogen Receptor				
Negative	1		1	
Positive	0.53 (0.46–0.61)	< 0.001	0.86 (0.67–1.10)	0.218
Unknown	0.85 (0.67–1.08)	0.195	2.46 (0.60–10.15)	0.212
Progesterone Receptor				
Negative	1		1	
Positive	0.56 (0.48–0.65)	< 0.001	0.96 (0.76–1.20)	0.724
Unknown	0.92 (0.73–1.16)	0.484	0.31 (0.08–1.27)	0.103
HER2^a^				
Negative	1		Not included	
Positive	0.83 (0.57–1.22)	0.347		
Borderline/Unknown	1.20 (0.87–1.66)	0.266		
Surgery				
Breast-conserving surgery	1		1	
Mastectomy	2.22 (1.90–2.59)	< 0.001	1.26 (1.04–1.54)	0.02
Others/Unknown	7.05 (5.77–8.62)	< 0.001	6.57 (5.04–8.55)	< 0.001
Radiation therapy				
No	1		1	
Yes	0.54 (0.47–0.62)	< 0.001	0.92 (0.77–1.10)	0.351
Unknown	0.50 (0.24–1.05)	0.067	0.54 (0.25–1.17)	0.12
Chemotherapy				
No	1		1	
Yes	0.89 (0.77–1.03)	0.116	0.67 (0.55–0.80)	< 0.001
Unknown	0.72 (0.53–0.99)	0.043	0.61 (0.43–0.85)	0.004
Endocrine therapy				
No	1		1	
Yes	0.44 (0.38–0.51)	< 0.001	0.60 (0.50–0.73)	< 0.001
Unknown	0.56 (0.40–0.78)	< 0.001	0.77 (0.54–1.09)	0.145

*HR* Hazard ratio, *CI* confidence interval

^a^Only patients diagnosed after 2010 were included

**Fig. 1:**
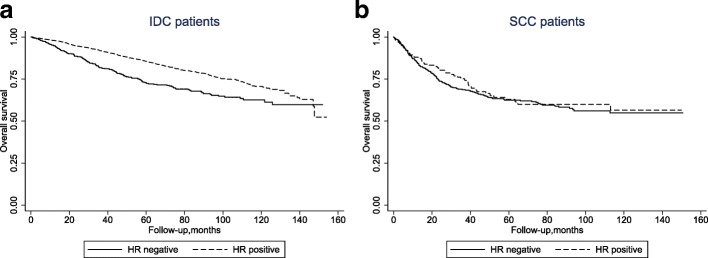
Kaplan-Meier survival analysis stratified by HR status in (**a**) IDC and (**b**) SCC patients

**Fig. 2:**
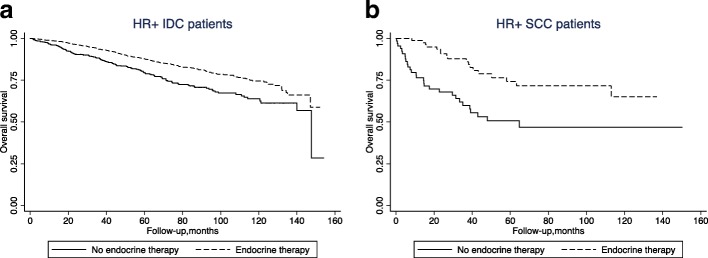
Kaplan-Meier survival analysis stratified by endocrine therapy in (**a**) HR-positive IDC patients, and (**b**) HR-positive SCC patients

We hypothesized that there was interaction between hormonal status (positive vs. negative) and the histology (SCC vs. IDC) in the analysis of OS. We performed a subgroup analysis and noticed that positive (vs. negative) hormonal status was significantly associated with improved OS in IDC (*P* < 0.01) patients, but not in the SCC (*P* = 0.042) patients (Fig. 1, Interaction test, *P* = 0.023). However, endocrine therapy was also associated with improved OS for the HR-positive IDC patients (HR = 0.61, 95%CI 0.48–0.77, *P* < 0.001), as well as for the HR-positive SCC patients (HR = 0.30, 95%CI 0.15–0.59, *P* < 0.001) (Fig. 2), after adjusting for age, race, comorbidity score, grade, T-stage, N-stage, M-stage, and chemotherapy.

## Discussion

### Prognosis of SCC patients

Previous studies have shown that SCC of the breast is more likely to be HR-negative and is associated with worse clinical outcomes [2, 8, 9]. Hennessy et al. [2] reported that the 5-year overall survival (OS) rates of 33 and 137 SCC patients selected from the M.D. Anderson Cancer Center and the SEER database, respectively, were 40 and 64%, respectively. In an update, Yadav et al [9] reported a 5-year cancer-specific survival rate of 63.5% for 445 SCC patients who were collected from the SEER database. Due to the rarity of SCC, only the data from the national cancer database is able to provide adequate statistical power to study the prognosis of SCC. However, several important prognostic factors, and the information of adjuvant therapies were lacking in the SEER database. In contrast, the NCDB database provides more prognostic factors (e.g comorbidity score, HER2 status and LVI status), as well as the information of adjuvant therapies (chemotherapy and endocrine therapy), therefore allows a more accurate estimation with less bias. In this study, we observed that the SCC patients had poorer clinicopathological features (e.g., T3–4, grade III, HR-negative disease) than the IDC patients. The adjusted analysis demonstrated that the SCC patients had significantly worse clinical outcomes than the IDC patients in both the 2004–2014 and 2010–2014 (HER2 status adjusted) cohorts. In consistent with previous studies, we confirmed that SCC (vs. IDC) is associated with poorer OS, after adjusting for more prognostic factors and adjuvant therapies.

### SCC & systemic therapies

The concept that SCC has worse clinical outcomes than IDC is likely to affect the choice of treatments in the clinical practices. This is confirmed in our study that chemotherapy was more prevalent in low-risk (HR+/HER2-/LN-) SCC (vs. IDC) patients (42.9% vs. 18.7%, *P* = 0.002). However, whether the SCC is responsive to chemotherapy is still unknown. Hennessy et al. [2] and Zhang et al. [6] reported that they used no responses were observed in their SCC patients after neoadjuvant chemotherapy. Only two case-reports [10, 11] have suggested that cisplatin-based chemotherapy is able to achieve long-term control, but these results need further verification. In our study, we noticed that SCC (vs. IDC) was significantly associated with no responses to neoadjuvant chemotherapy (20% vs. 5.05%, *P* = 0.019). Therefore, the benefit of chemotherapy in SCC patients remains unknown.

In this study, we observed that the endocrine therapy was less performed in the HR-positive SCC (51.6%) patients, than in the HR-positive IDC (70.5%) patients. A contributing reason could be that endocrine therapy in the head & neck or esophageal SCC patients is not useful in clinical practices [12, 13], even if the in vitro evidences [14, 15] had suggested the role of tamoxifen for SCC of the oral cavity or esophagus. However, SCC of the breast might possibly be different from the head & neck SCC. A recent study used whole-exome sequencing to show that the SCC components have nearly identical landscapes of somatic mutations to their adjacent IDC component, suggesting that SCC may originate from the IDC [3]. Since the role of endocrine therapy had been established in IDC patients, it is possible the endocrine therapy would also be beneficial in SCC patients. In the analysis of prognostic factors of OS, we observed a significant interaction between the HR status and the histology (SCC vs. IDC). The HR status was prognostic only for IDC patients, but not for SCC patients. Despite of this, we still observed that the endocrine therapy significantly improves the OS in HR-positive IDC and SCC patients. Taken together, we suggested that endocrine therapy should remain as the standard treatment for HR-positive SCC patients. Ng et al. [5] studied the landscape of somatic genetic alterations of SCC and reported that TP53(78%) and PI3KCA(44%) are the most frequently mutated genes in SCC. They proposed that the mutation affecting genes might result in the Wnt and mTOR pathway activation. Future studies are warranted to investigate whether relevant pathway inhibitors could be used for SCC patients.

### SCC & local therapy

We assessed the influence of the SCC component on the local therapy. SCC patients had a slightly lower rate of BCS, when compared to IDC patients (58.3% vs. 65.4%, *P* = 0.048). The underlying reason could be that the SCC patients had larger tumor than IDC patients (T3–4: 23.1% vs. 4%). Among patients with BCS, RT was used in 65.3 and 83.0% of the SCC and IDC patients, respectively. Currently, evidence that support the use of RT for SCC is lacking. Hennessy et al. [2] reported that 4 out of 19 SCC patients treated with RT had locoregional relapse within the irradiated field, suggesting that SCC might be radioresistant. Two studies [16, 17] reported no benefit of RT on OS, but the small sample sizes in these studies limited their statistical power. Using the SEER database, Wu et al. [18] reported that RT was significantly associated with improved OS but not cancer-specific survival, which is difficult to explain. Furthermore, they reported that RT was significantly associated with improved CSS in stage II SCC patients, but the analysis was not adjusted for ER, PR or HER2. Thus, the role of RT as an adjuvant local control therapy after surgery remains controversial.

### Limitations

First, it is possible that the IDC patients may have a small proportion of SCC component area. Without pathological confirmation, grouping the cases into IDC, and SCC might not be always accurate. However, a detailed histopathology examination is impossible in mining large database, such as NCDB. The large sample size of this study population is able to compensate this limitation. Second, nonrandomized comparisons of treatment effects are prone to providing misleading estimations. One study [19] showed that the treatment effect of RT in breast cancer was over-estimated in observational data compared with randomized clinical trial data. This effect is reasonable as the “treatment-by-indication” bias can never be eliminated in observational data. Therefore, we did not estimate the survival benefit of chemotherapy, as patients with more advanced diseases are prone to receive chemotherapy. However, estimation of the survival benefit of endocrine therapy in HR-positive patients is less likely to be affected by the “treatment-by-indication” bias. HR-positive is the only indication for endocrine therapy. In addition, the decision to implement the endocrine therapy is less likely to be influenced by the comorbidity status. Thus, estimation of the survival benefit of endocrine therapy using observational data is reasonable. Due to the rarity of SCC of the breast, a prospective, randomized study for SCC cannot be realistically implemented. Third, the NCDB did not have information regarding trastuzumab therapy. Whether trastuzumab would be appropriate for HER2-positive SCC patients remains unclear. The lack of information regarding local relapse, metastatic relapse and cancer-specific survival is also one of the limitations. Fourth, there is a growing awareness of an association between SCC of the breast and implants. But the significance of the association is unclear due to the rarity of this situation [20–22]. The NCDB database does not have the information about the history of breast implants augmentation before the diagnosis of SCC, therefore limits our understanding about this issue.

## Conclusions

In this study, we show that compared with IDC, SCC is associated with poorer clinicopathological outcomes. The treatment patterns differ between IDC and SCC. Radiotherapy after BCS is used less often for SCC (vs. IDC) patients. Chemotherapy is used more often for low-risk (HR+/HER2-, node-negative) SCC (vs. IDC) patients. Endocrine therapy is used less often in HR-positive SCC (vs. IDC) patients. In addition, SCC is less likely to response to chemotherapy, and is associated with worse clinical outcomes. Although the HR status is not prognostic in SCC patients, endocrine therapy is still associated with improved OS in HR-positive SCC patients.

## Additional file

Additional file 1: **Figure S1.** Kaplan-Meier survival analysis stratified by histology (IDC vs. SCC) and nodal status in a) HR-positive and b) HR-negative patients. (PDF 62 kb)

## Abbreviations

BCS: Breast-conserving surgery
ER: Estrogen receptor
HR: Hormonal receptor
IDC: Infiltrating ductal carcinoma
LN: Lymph node
LVI: Lymphovascular invasion
NCDB: National cancer database
OS: Overall survival
PR: Progesterone receptor
RT: Radiation therapy
SCC: Squamous cell carcinoma
