# Supplementary figures and images for: Clinicopathological features, treatment patterns, and prognosis of squamous cell carcinoma of the breast: an NCDB analysis
Source: BMC Cancer. 2019 Jan 8;19:26. doi: 10.1186/s12885-018-5212-x (PMC6323666; doi:10.1186/s12885-018-5212-x)

# Supplemental Figure 1

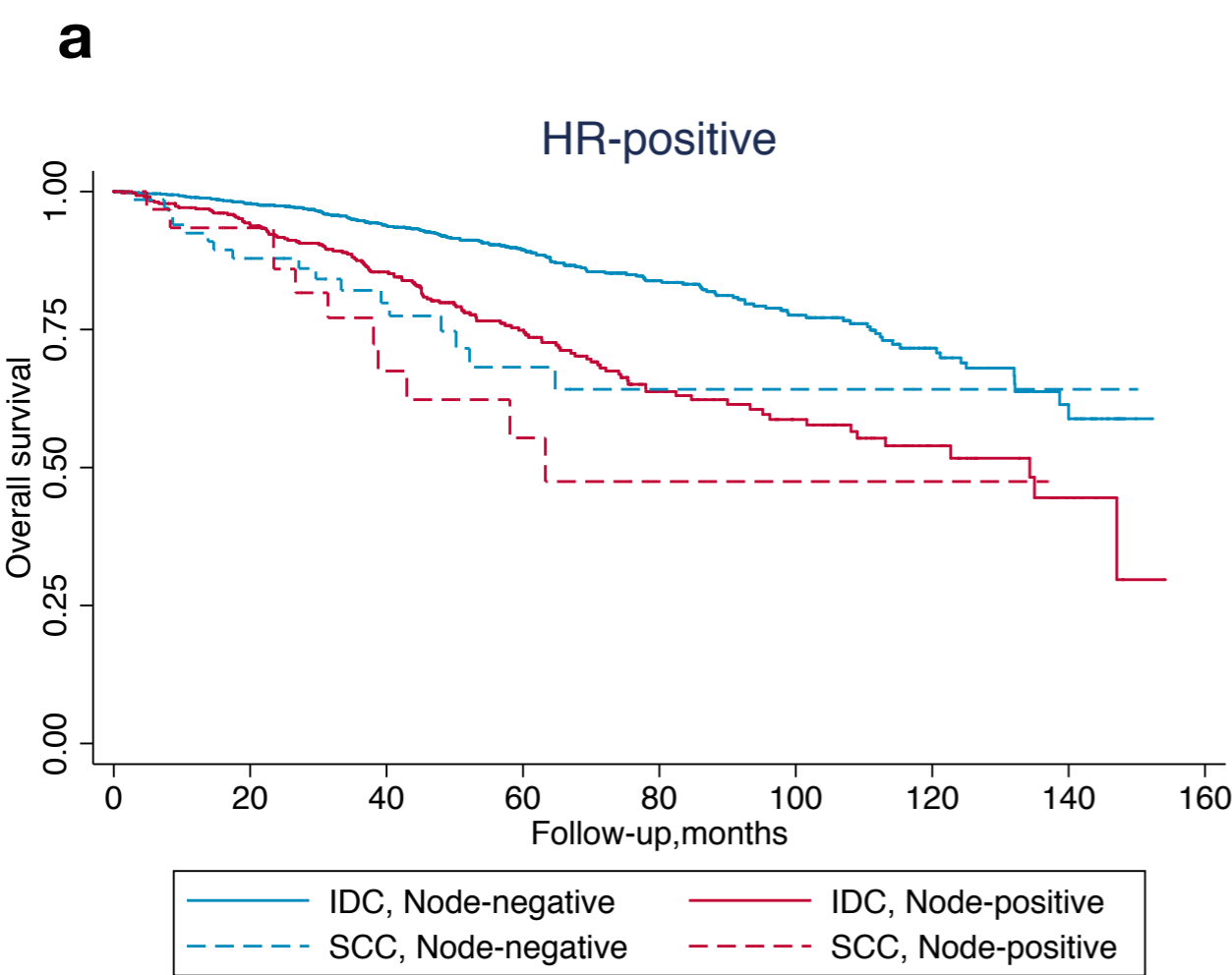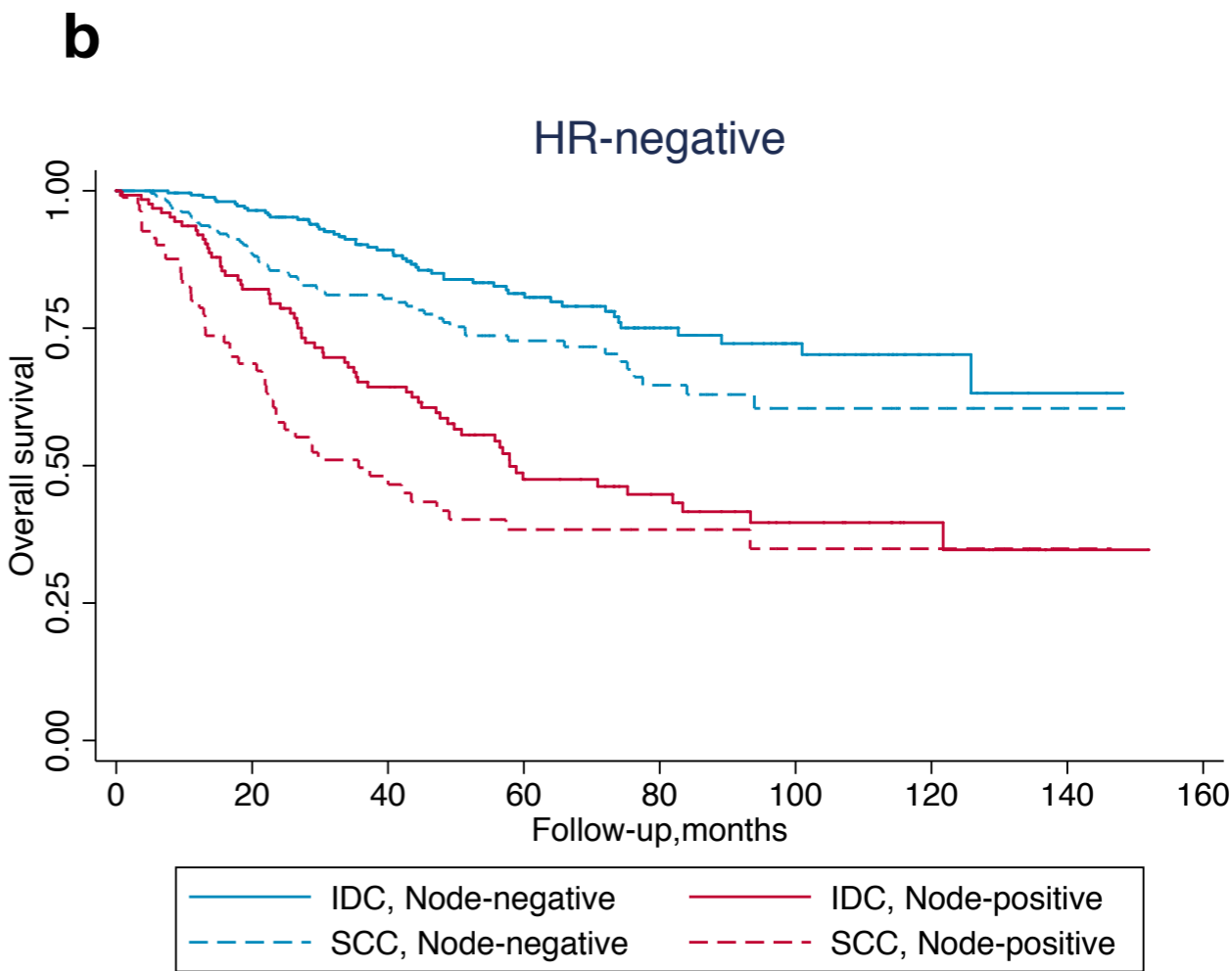

Supplement: Supplementary file 1 — Figure S1. Kaplan-Meier survival analysis stratified by histology (IDC vs. SCC) and nodal status in a) HR-positive and b) HR-negative patients. (PDF 62 kb) [file 12885_2018_5212_MOESM1_ESM.pdf]
